# Supplementary figures and images for: The temporal regulation of TEK contributes to pollen wall exine patterning
Source: PLoS Genet. 2020 May 14;16(5):e1008807. doi: 10.1371/journal.pgen.1008807 (PMC7252695; doi:10.1371/journal.pgen.1008807)

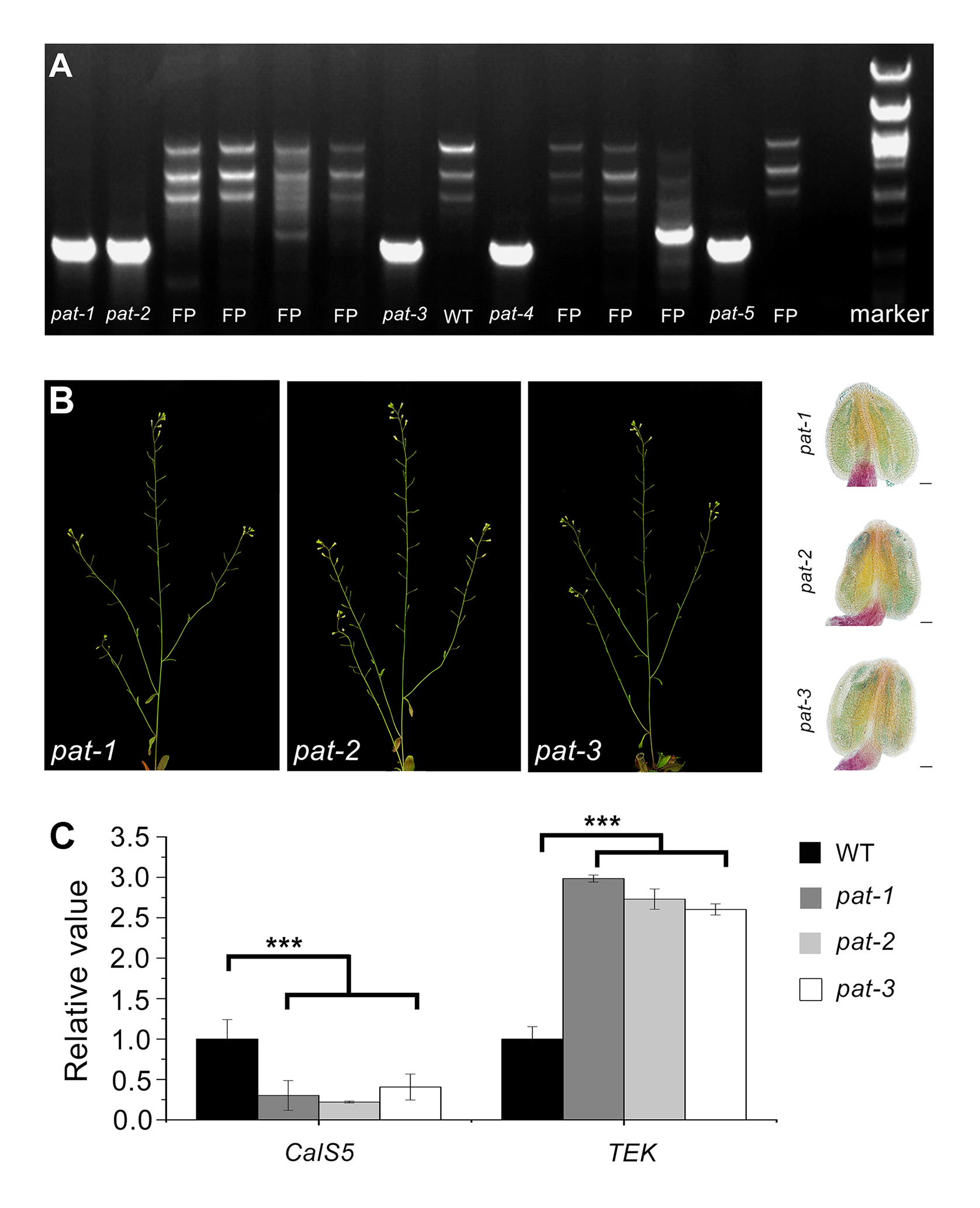

Supplement: S1 Fig — (A) In the T1 generation, the presence of insertion in independent pat transgenic plants was confirmed by PCR. A 694-bp DNA including the AMS promoter and TEK genomic fragment was amplified using primers PAMSJD-F and CTEKJD-R. The plants without the insertion of the target fragment were fertile and were named FP (Fertile Plants). (B) Three independent pat transgenic lines are shown, and they are all male sterile, as confirmed by Alexander’s staining of anthers. Scale bars, 20 μm. (C) Expression of CalS5 and TEK was detected in three independent pat lines by qRT-PCR analysis. Error bars represent the SD (n = 3). *** p < 0.001 (t-test). (TIF) [file pgen.1008807.s001.tif]

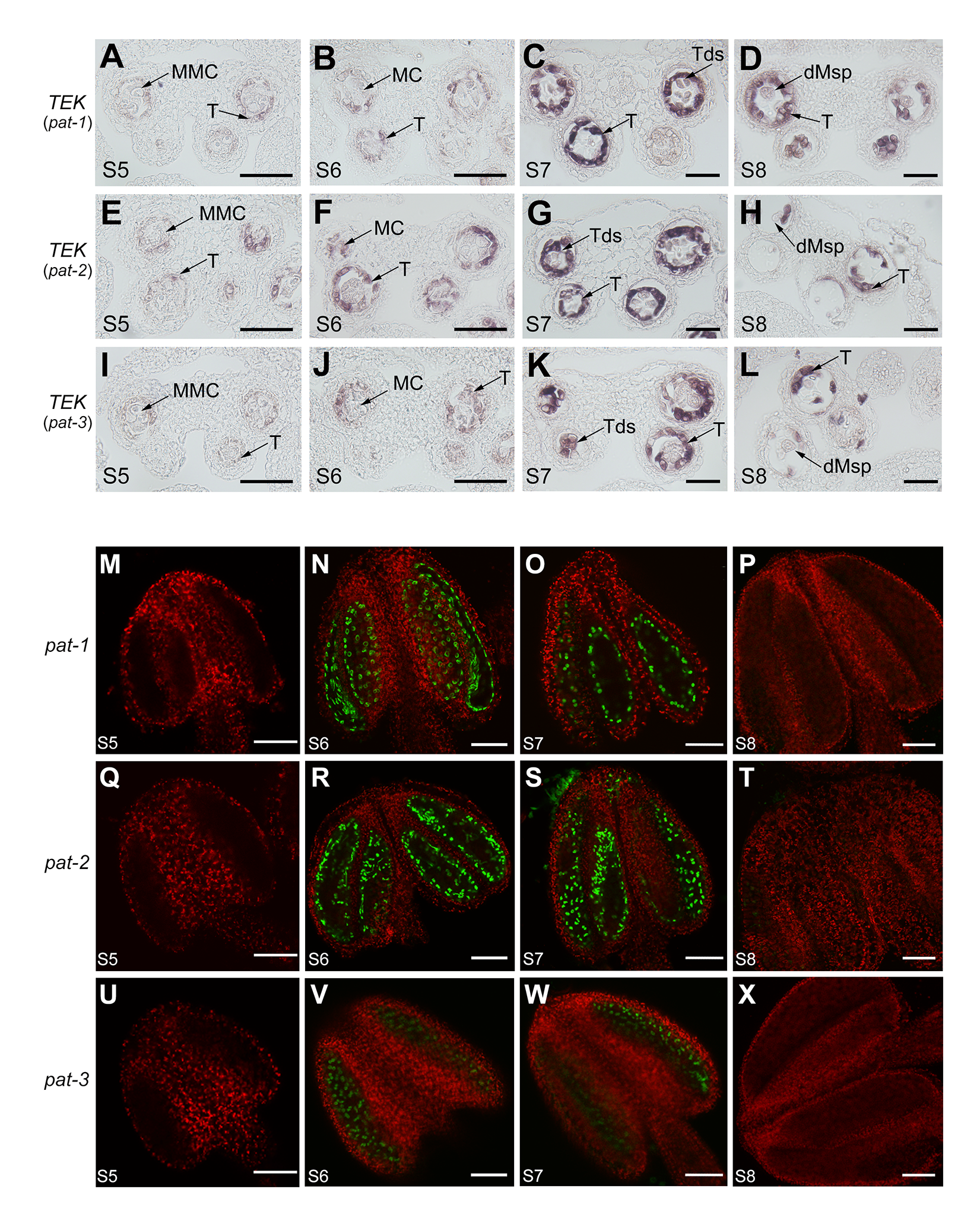

Supplement: S2 Fig — RNA in situ hybridization of TEK transcripts in anthers of pat-1 (A–D), pat-2 (E–H) and pat-3 (I–L) at stages 5–8 using an antisense probe. MMC, microspore mother cell; MC, meiocytes; T, tapetum; Tds, tetrads; dMsp, degenerated microspore. Scale bars, 20 μm. Fluorescence confocal images of the TEK–GFP fusion protein in anthers of pat-1 (M-P), pat-2 (Q-T) and pat-3 (U-X) at stages 5–8. Scale bars, 50 μm. (TIF) [file pgen.1008807.s002.tif]

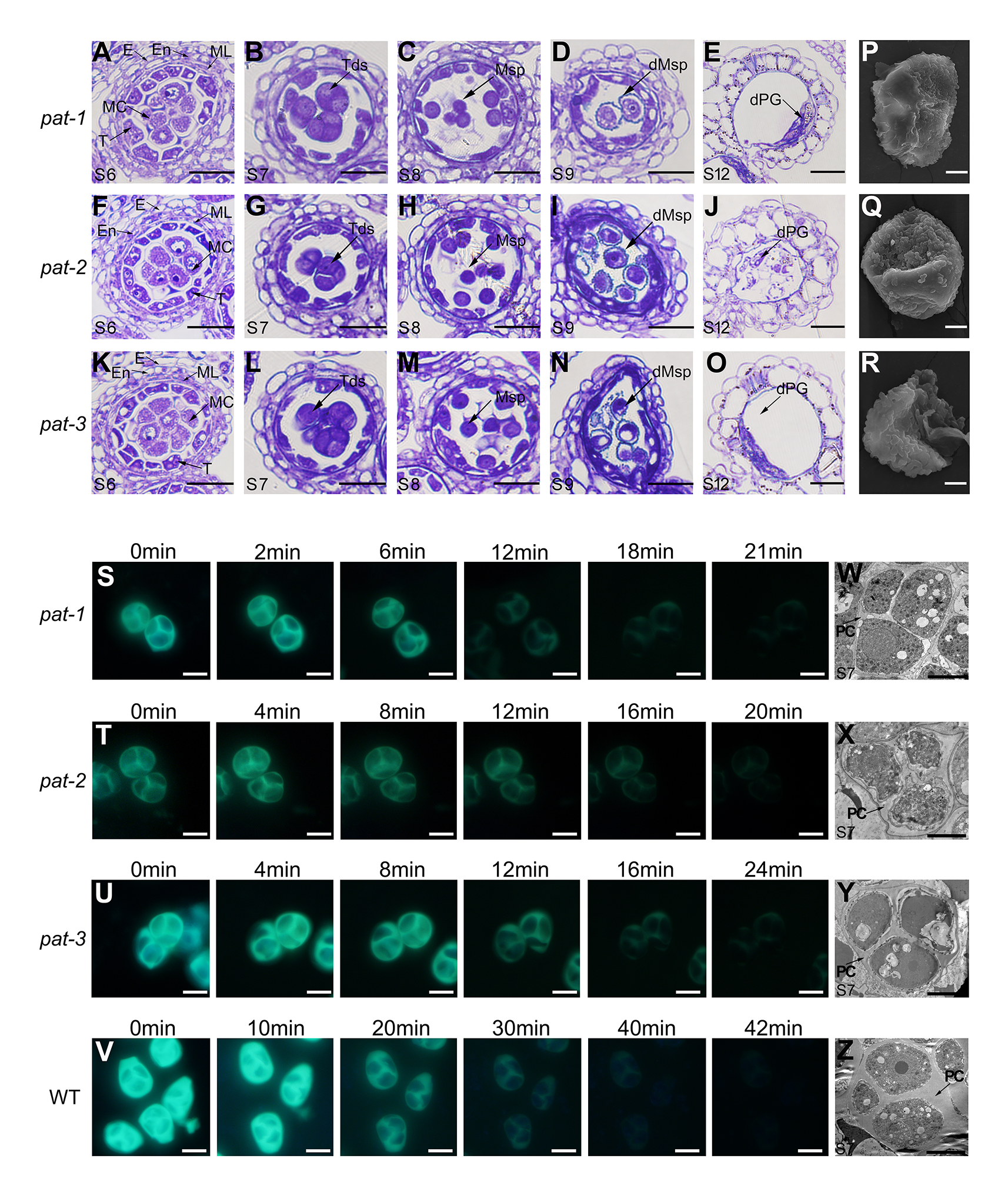

Supplement: S3 Fig — Semi-thin sections of pat-1 (A-E), pat-2 (F-J) and pat-3 (K-O) showing anther development from stages 6–12. E, epidermis; En, endothecium; ML, middle layer; T, tapetum; MC, meiocytes; Tds, tetrads; Msp, microspore; dMsp, degenerated microspore; dPG, degenerated pollen grains. Scale bars, 5 μm. SEM observation of pollen grains in pat-1 (P), pat-2 (Q) and pat-3 (R). Scale bars, 10 μm. The callose fluorescence quenching assay showed that callose wall fluorescence in pat-1 (S), pat-2 (T) and pat-3 (U) quenched faster than that in WT (V). Scale bars, 20 μm. TEM observation of tetrads in pat-1 (W), pat-2 (X) and pat-3 (Y) at stage 7 compared with that in WT (Z). PC, peripheral callose. Scale bars, 2 μm. (TIF) [file pgen.1008807.s003.tif]

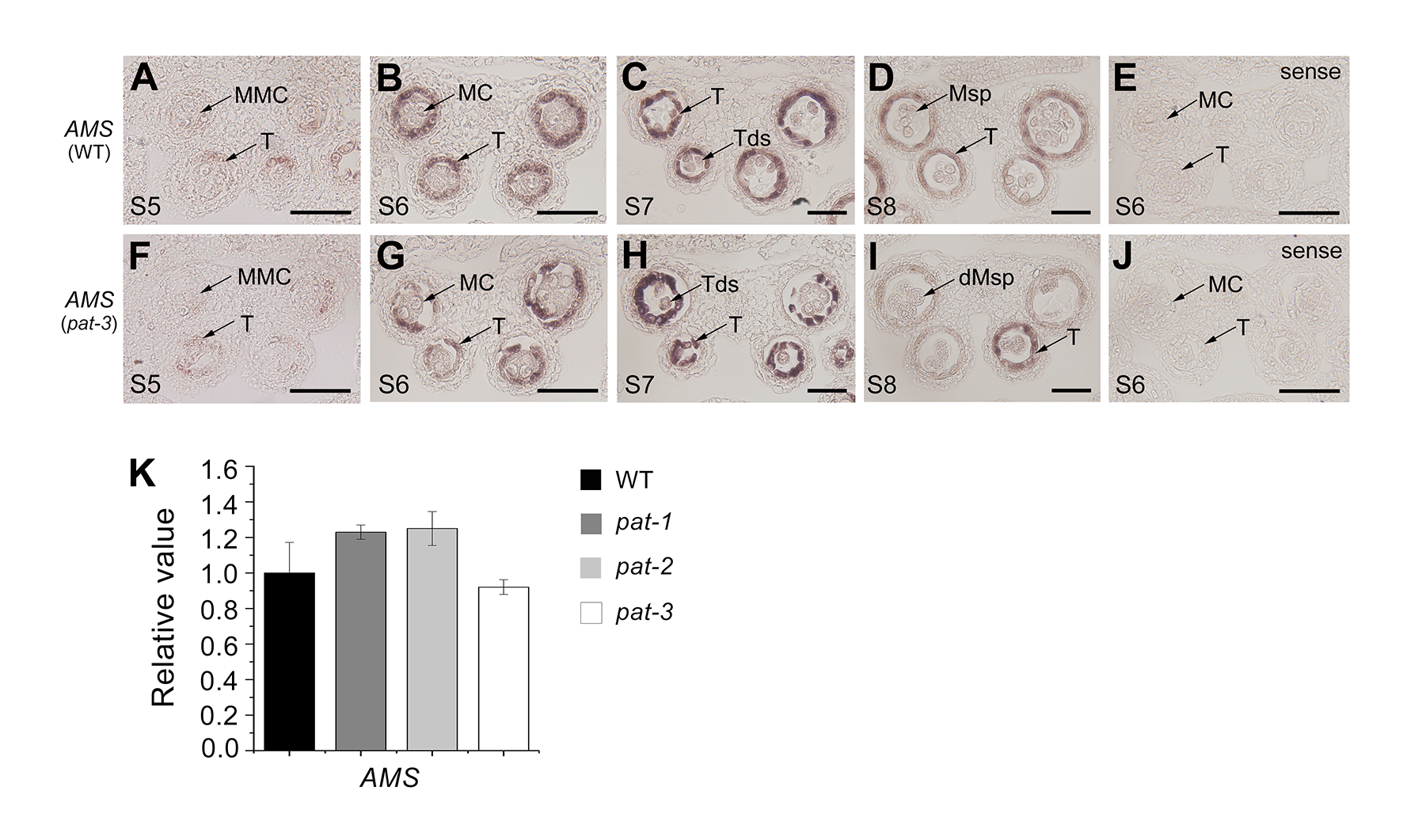

Supplement: S4 Fig — RNA in situ hybridization of AMS transcripts in the anthers of WT (A–D) and pat-3 (F–I) at stages 5–8 using an antisense probe. AMS transcript in anthers of WT (E) and pat-3 (J) using a sense probe at stage 6. MC, meiocytes; T, tapetum; Tds, tetrads; Msp, microspore; dMsp, degenerated microspore. Scale bars, 20 μm. (K) Expression of AMS was detected in three independent pat lines by qRT-PCR analysis. Error bars represent the SD (n = 3). (TIF) [file pgen.1008807.s004.tif]

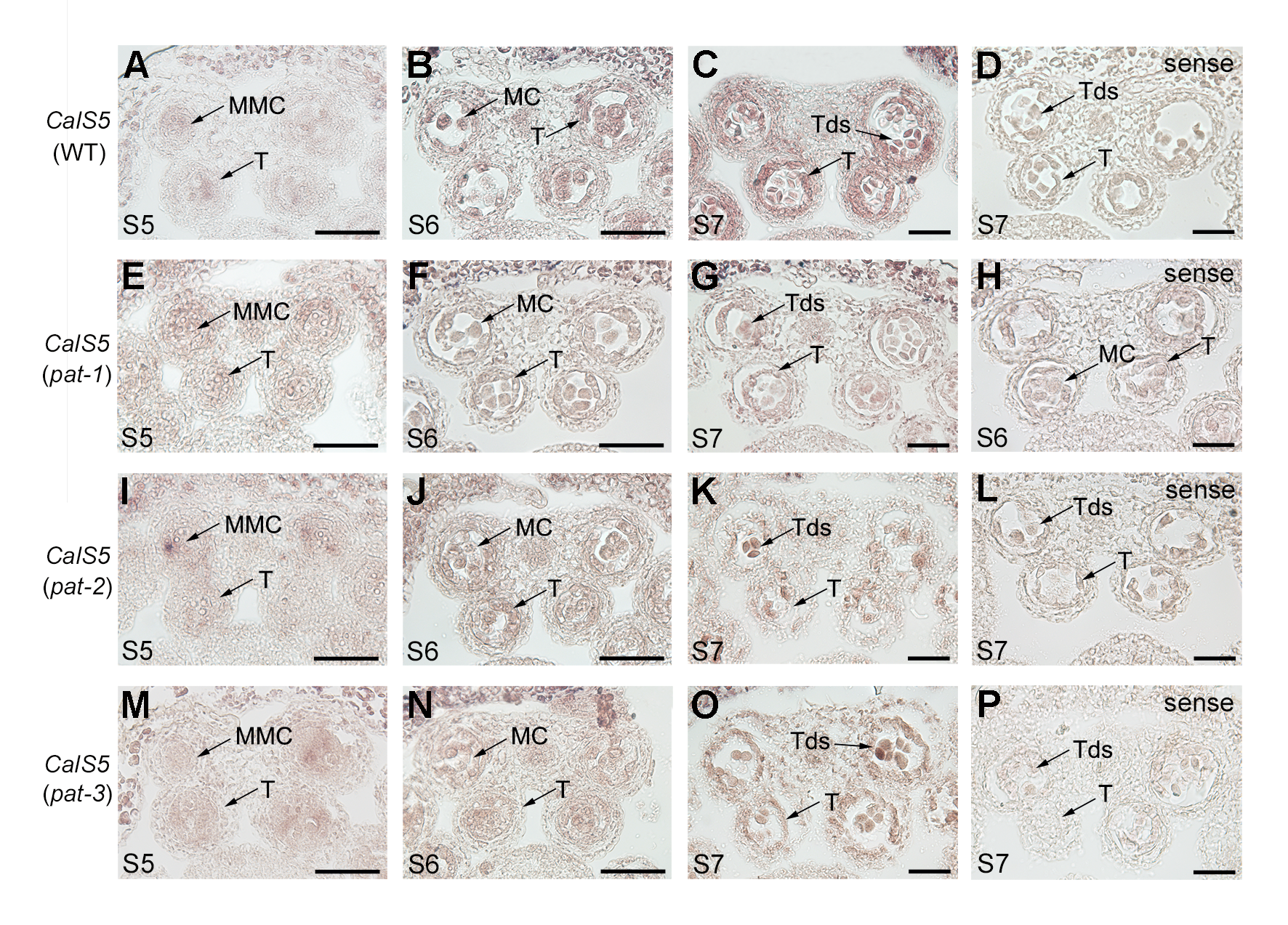

Supplement: S5 Fig — Expression of CalS5 in microspore mother cells, tetrads and tapetum was detected by RNA in situ hybridization in anthers of WT (A–C), pat-1 (E-G), pat-2 (I-K) and pat-3 (M-O) at stages 5–7 using an antisense probe. CalS5 transcript in WT (D) and pat anthers (H, L, P) using a sense probe. MMC, microspore mother cell; MC, meiocytes; T, tapetum; Tds, tetrads. Scale bars, 20 μm. (TIF) [file pgen.1008807.s005.tif]

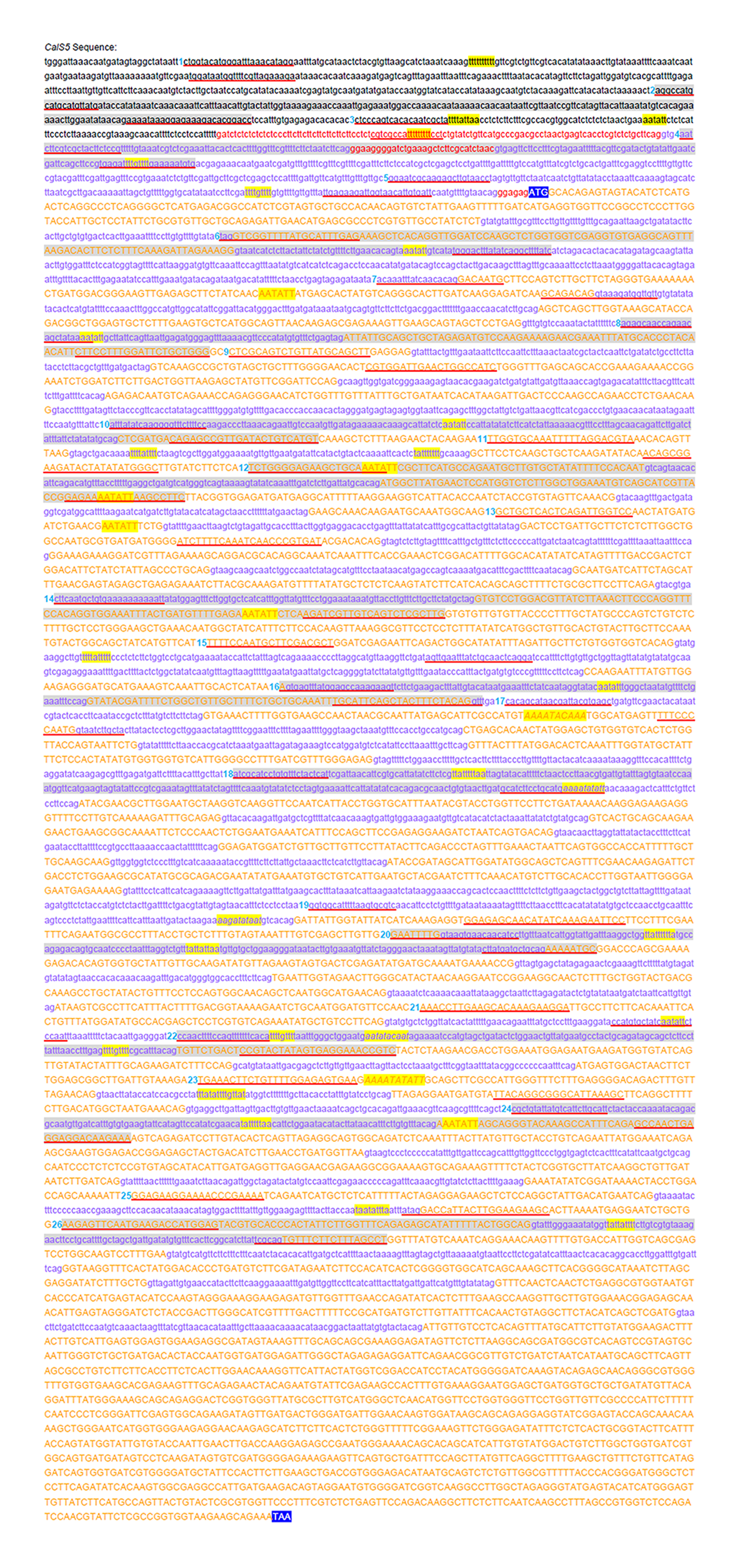

Supplement: S6 Fig — There are 26 pairs of primers for ChIP marked by blue serial numbers. The text highlighted in yellow indicates the AT-rich sequences. Underlined text indicates the detailed locations of primers. (TIF) [file pgen.1008807.s006.tif]

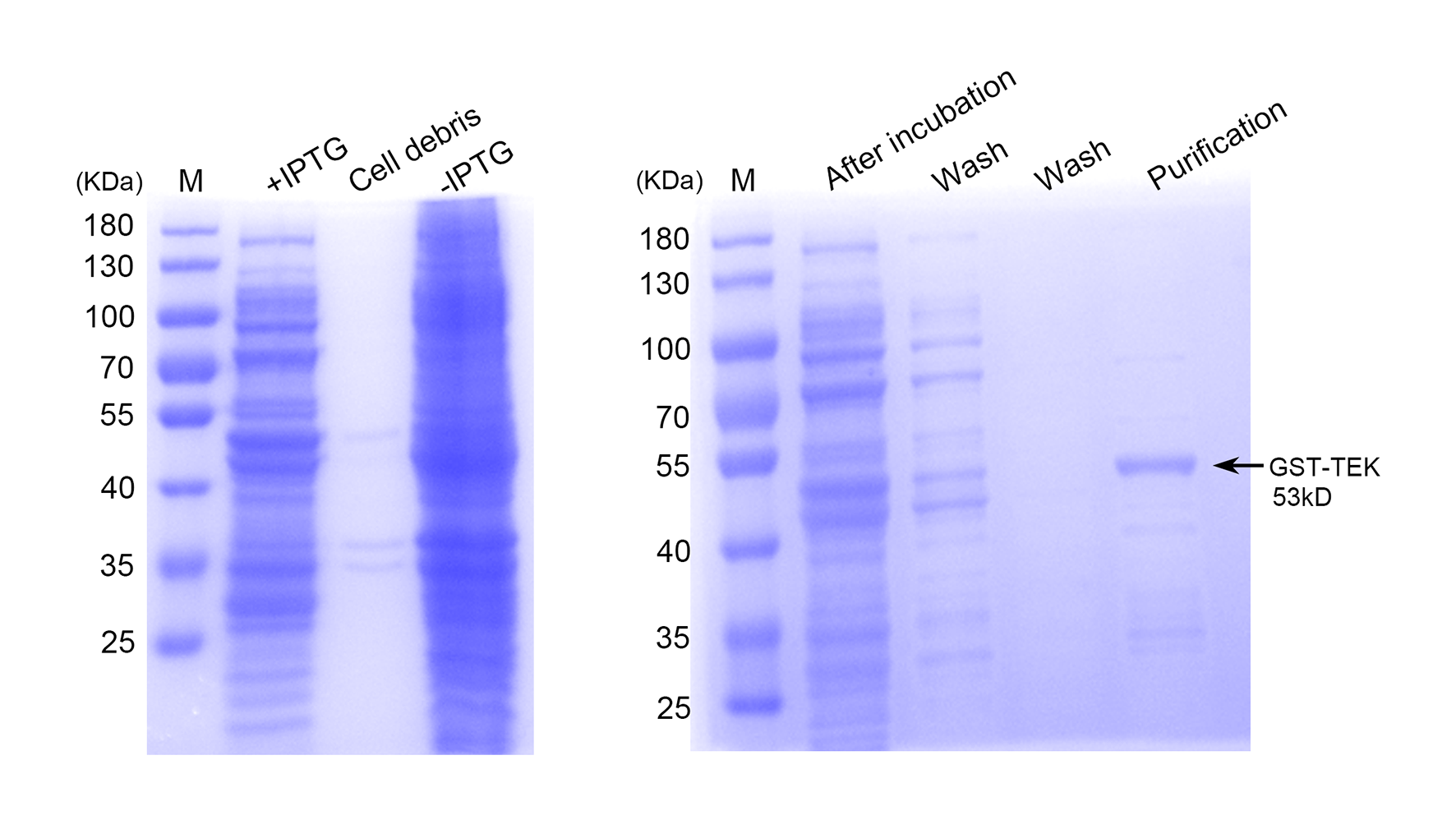

Supplement: S7 Fig — SDS-PAGE analysis of GST-TEK proteins used for in vitro EMSA analysis. Purified proteins were run on an 8% gradient gel and stained with Coomassie blue. M, protein markers. (TIF) [file pgen.1008807.s007.tif]
