# Supplementary material for: The temporal regulation of TEK contributes to pollen wall exine patterning
Source: PLoS Genet. 2020 May 14;16(5):e1008807. doi: 10.1371/journal.pgen.1008807 (PMC7252695; doi:10.1371/journal.pgen.1008807)
Supplement: Attachment — Submitted filename: response to reviewers conmments.docx [file pgen.1008807.s010.docx]

Dear Dr Lou,

Thank you very much for submitting your Research Article entitled 'The temporal regulation of TEK contributes to pollen wall exine patterning' to PLOS Genetics. Your manuscript was fully evaluated at the editorial level and by independent peer reviewers. The reviewers appreciated the attention to an important problem, but raised some substantial concerns about the current manuscript. Based on the reviews, we will not be able to accept this version of the manuscript, but we would be willing to review again a much-revised version. We cannot, of course, promise publication at that time.

Should you decide to revise the manuscript for further consideration here, your revisions should address the specific points made by each reviewer and the guest editor. We will also require a detailed list of your responses to the review comments and a description of the changes you have made in the manuscript.

If you decide to revise the manuscript for further consideration at PLOS Genetics, please aim to resubmit within the next 60 days, unless it will take extra time to address the concerns of the reviewers, in which case we would appreciate an expected resubmission date by email to plosgenetics@plos.org.

If present, accompanying reviewer attachments are included with this email; please notify the journal office if any appear to be missing. They will also be available for download from the link below. You can use this link to log into the system when you are ready to submit a revised version, having first consulted our Submission Checklist.

To enhance the reproducibility of your results, we recommend that you deposit your laboratory protocols in protocols.io, where a protocol can be assigned its own identifier (DOI) such that it can be cited independently in the future. For instructions see our guidelines.

Please be aware that our data availability policy requires that all numerical data underlying graphs or summary statistics are included with the submission, and you will need to provide this upon resubmission if not already present. In addition, we do not permit the inclusion of phrases such as "data not shown" or "unpublished results" in manuscripts. All points should be backed up by data provided with the submission.

While revising your submission, please upload your figure files to the Preflight Analysis and Conversion Engine (PACE) digital diagnostic tool. PACE helps ensure that figures meet PLOS requirements. To use PACE, you must first register as a user. Then, login and navigate to the UPLOAD tab, where you will find detailed instructions on how to use the tool. If you encounter any issues or have any questions when using PACE, please email us at figures@plos.org.

PLOS has incorporated Similarity Check, powered by iThenticate, into its journal-wide submission system in order to screen submitted content for originality before publication. Each PLOS journal undertakes screening on a proportion of submitted articles. You will be contacted if needed following the screening process.

To resubmit, use the link below and 'Revise Submission' in the 'Submissions Needing Revision' folder.

We are sorry that we cannot be more positive about your manuscript at this stage. Please do not hesitate to contact us if you have any concerns or questions.

Yours sincerely,

Anna A. Dobritsa

Guest Editor

PLOS Genetics

Gregory Copenhaver

Editor-in-Chief

PLOS Genetics

Guest editor's comments:

Both reviewers conclude - and I agree with their assessment - that the findings are potentially interesting but you will need more evidence to demonstrate that male sterility in pat plants is indeed caused by the mechanism which you propose: that the premature expression of TEK in tapetum leads to problems with the formation of the callose wall around microspores and that TEK directly represses the callose synthase CalS5 gene by interacting with its regulatory regions and causing the appearance of repressive histone marks. As the analysis of a single transgenic line cannot be considered sufficient in this case (the effects could be caused by the position of the transgene insertion), you would need to perform the critical experiments which led to these conclusions on at least two more independent transgenic lines. This is particularly important because the mechanism that you propose is not consistent with the more accepted idea that microsporocytes themselves synthesize their callose walls. Therefore, you need to ensure that your conclusions, which imply that a lot of CalS5 activity comes from tapetum, are very strongly supported by experimental evidence.

Answer: We appreciate the editor’s precious suggestions to this work. The major concern from editor and reviewers is that more independent *pat* lines should be tested including the cytological observations, the TEK expression timing and *CalS5* expression to ensure the phenotype we have observed doesn’t come from the position of the transgene insertion in an individual transgenic line. Actually, in this work, we first transformed the *pAMS:TEK-GFP* construct into the WT plants, finding that T1 generation lines are all male sterile only they contain the insertion by PCR identification (Fig. S1A). We then repeated this transformation several times, and found the T1 generation with transgene insertion all showed the male sterile phenotype. We also examined the independent *pat* lines in T1 generation, finding that TEK expresses earlier and expression of *CalS5* is also reduced in each line (Figs. S1 and S2). Additionally, we used WT pollen to cross-fertilize several independent *pat* stigmas for producing their F1 seeds. In these F1 generation, we used PCR to confirm again that plants show male sterility with insertion while others show normal without insertion. Therefore, we think the phenotype in *pat* lines did result from the premature expression of TEK in tapetum and we uniformly name them as *pat*. However, because we didn’t show these data in the former version which confused you and let you misunderstand our data just from one single transgenic line. We feel very sorry about that! In this version, we provided all the experiment data about three individual *pat* lines as editor and reviewers required (Figs. S1-S5).

In addition to providing detailed answers to the questions and concerns raised by the reviewers, I would encourage you to provide more evidence of the following:

1. The callose wall defects and the timing of their appearance in pat lines. The callose wall defects are far from obvious. There is little difference in the appearance of the callose wall in WT and in pat in the light-microscopy images (Figs. 1H and 1N, Figs. S2A and S2B). Even in TEM images (Figs. 3L and 3Q), for which you state that the “callose wall was obviously sparse”, this is not at all obvious and requires some strong quantitative support.

Answer: We admitted that the reduction of *pat* callose wall is not so obvious than other mutants with severely defects such as *cals5* and *arf17*. However, we confirmed that the peripheral callose wall is relatively thinner than that in WT (Fig. 2Q and 2R). It has been reported that callose synthesis is impaired in *npu* and *cdkg1* mutants (Chang et al., 2012; Huang et al., 2013). We found that the phenotype of *pat* callose wall by aniline blue staining is similar with those of *npu* and *cdkg1*. The reduction expression of *CalS5* in *pat* is also similar with that in *npu* and *cdkg1.* Moreover, the abnormal appearance of *pat* callose wall can be found in Fig. 4C. As to the TEM results, the density of callose wall begins to be reduced at early stage 7 (Fig. 3Q). If you enlarge the images, it can be found that the *pat* callose wall is loose while that of WT is compact without lacuna (Fig. 3G). Later, the callose wall in *pat* is thinner than that in WT (Fig. 3R and 3S).

Even so, we really pay attention to the editor’s opinion. First, we modified our language to better describe our results. Then, we provided the TEM imagines of *pat-1,* *pat-2* and *pat-3* at stage 7 (Fig. S3W-Y), in which the sparse callose wall can be easier found. In the bleaching test of callose ﬂuorescence, their callose wall also quenched faster than that in WT (Fig. S3S-V). So, all these results show that callose wall becomes thinner, callose synthesis is reduced and this defect initiates at tetrad stage in *pat* lines.

2. Timing of TEK expression has to be tested in additional pat lines.

Answer: We provided imagines of TEK expression in three independent *pat* lines and the results showed that the fluorescence of TEK-GFP also early expresses at stage 6 (Fig. S2N, R, V). Moreover, we also detected the *TEK* expression pattern in these three lines (Fig. S2A-L) and the results are similar with Fig. 1G-J. Therefore, we believe that TEK did precociously express in tapetum in *pat* transgenic lines.

3. Reduction of Cals5 expression has to be verified in additional pat lines, and I would suggest that this should be also demonstrated with methods other than just qRT-PCR.

Answer: We provided the data of *CalS5* expression in *pat-1, pat-2* and *pat-3* lines by qRT-PCR, showing that the *CalS5* expression is largely reduced in each line (Fig. S1C). Furthermore, we performed *in situ* hybridization in the above lines, showing that the *CalS5* transcripts are also reduced in these anthers (S5 Fig).

4. The binding of TEK to the P11 region and the enrichment of H3K9Me2 in this region has to be tested in additional pat lines. Further, the ability of TEK to bind to this region has to be confirmed by an independent method, such as EMSA. Please also provide more information on the position of the P11 region in relation to the CalS5 gene model (e.g. Is it in an intron? In which one?) as well as the details for the qPCR experiments performed in conjunction with the ChIP experiments. Fig. 6A shows 26 regions that were supposedly tested by ChIP-qPCR, yet the graphs in Figs. 6B and C show the results from much fewer regions. What was done on the other regions? Also, to be confident in results of qPCR, they have to be performed very carefully – e.g. all primers have to be tested for their ability to double the amount of DNA at each cycle and Ct values have to be in a certain range to be reliable. Please provide evidence that all the proper controls were done for each tested region and the results were indeed reliable. Please add the supplemental table with all the primers (it seems to be missing from the submission).

Answer: In fact, we first took several T1 lines to detect their phenotypes, which all showed the similar results including the phenotype of male sterility, the premature TEK expression, the reduction of *CalS5* expression and the impaired callose synthesis (Figs. S1-3). Thus, we think the phenotype in *pat* lines did result from the premature expression of TEK in tapetum. Based on this conclusion, we then used WT pollen to cross-fertilize several independent *pat* stigmas for producing their F1 seeds. In these F1 generation, we used PCR to confirm again that plants show male sterility with insertion while others show normal without insertion. Therefore, we collected these inflorescences from the male sterile plants with transgene insertion due to ChIP assay requires lots of inflorescences. So, the materials for ChIP assay are the sets which consist of the male sterile plants with transgene insertion instead of the progeny from a single line. Additionally, we collected the ChIP materials three times from different DNA samples and qChIP-PCR were also performed two replications. In this version, we carefully modified the ChIP part in materials and methods, and we hope this explanation can clearly illuminate how we did the ChIP assay.

Using EMSA to confirm that TEK binds to the region amplified by primer set P11 is a good idea, which can strengthen our conclusion. Thus, we expressed the GST-TEK protein and purified this recombinant protein (S7 Fig). In the EMSA assay, we found that a specific bandshift resulted from the incubation between GST-TEK protein and P11 region. The reduced bandshift with the unlabeled probes and no bandshift in the negative group (Fig. 6C), further confirming the binding specificity.

In the ChIP assay, all primers we used were tested in control group and sample group. Then we calculated their △Ct value and took 2^-△Ct^ to obtain the fold enrichment. We think that this way can as much as possible to avoid the fold discrepancy derived from the DNA amplification efficiency of different primers. In this revision, we provided the detailed Ct values and all the ChIP-qPCR results in S2 Table. We also marked the detailed locations of primers connecting with the *CalS5* sequence in Fig. S6 and added the S1 Table for primers list. Thanks for these good suggestions which really make our work more rigorous and reliable!

5. Fig. 5 describes potentially important results but is presented so inconspicuously that even one of the reviewers missed the fact that the bottom row shows the CalS5 in situ hybridization in the tek mutant, not in pat. I would encourage you to also provide the in situ hybridization data on the Cals5 expression from several pat lines (and perhaps compare it with the CalS5 expression in the TEKpro:TEK-GFP lines) to strengthen your conclusions.

Answer: In this version, we labeled the detailed probes and materials in Fig. 5 and Fig. 1. Also, we performed the *in situ* hybridization to detect the *CalS5* expression in independent *pat* lines as editor suggested. Compare with WT, we found that the hybridization signals from several *pat* lines were reduced in tapetum (S5E-P Fig).

6. Please explain what is shown in the eight unlabeled lanes in Fig. S1A and provide the figure legend for Fig. S1C.

Answer: We labeled these eight lanes which are WT plants without insertion and we provided the expression of *TEK* and *CalS5* in different *pat* lines in Fig. S1C with figure legends. Thanks!

7. Remove the results on MS188 presented in Fig. S3. It is distracting when they are suddenly mentioned in the Discussion without being presented in the Results.

Answer: We removed the results in former Fig. S3. Thanks!

Reviewer's Responses to Questions

Comments to the Authors:

Please note here if the review is uploaded as an attachment.

Reviewer #1: The Manuscript ‘The temporal regulation of TEK contributes to pollen wall exine patterning’ by Xiong et al. presents a series evidences to state the temporal expression of TEK is essential for pollen exine patterning. Previous studies have revealed that TEK is required for nexine formation and the tek mutant exhibited pollen nexine absence in Arabidosis. Here in this manuscript, the authors performed an earlier than normal expression of TEK (AMS::TEK-GFP, name as pat) in wild type anther and found the premature expression of TEK leads to completely male sterility; they employ cytological and molecular analyses to demonstrated that TEK physically interacts with the P11 site of CalS5 promoter and represses the CalS5 expression via H3K9me2.

Overall, this is an interesting story that provided a mechanism that how the tapetum stop the callose wall synthesis and promote the callose wall degeneration to achieve the microspores release and the pollen wall patterning. The technical quality of the study could however be improved. I summarize my comments and suggestions below:

We appreciate for the reviewer’s recognition for this work.

1. I would suggest to do phenotypic analyses with more than two independent pat alleles, as least in some of the phenotypic analyses. Although fertility and alexander staining anther from 3 alleles were presented in S1, further cytological observations results were provided only in one allele.

Answer: We do agree with reviewer’s opinion. In this work, we had detected the cytological observations in other *pat* lines. However, because we didn’t show these data in the former version, the results confused all reviewers. This is our mistake and we feel very sorry about that! In this version, we provided more cytological observations of three independent *pat* alleles, including the phenotype of male sterility, the premature TEK expression, the reduction of *CalS5* expression and the impaired callose synthesis (Figs. S1-5). All these results showed that the phenotype in *pat* lines did result from the premature expression of TEK in tapetum. Thanks for this precious suggestion!

2. On a more detailed level, the manuscript lack of experimental details. This is general, but I take several as example:

1) I could not find any indication of Figure S1C, in which relative expression of TEK in only one allele is shown.

Answer: This is our mistake that we missed the figure legends for Figure S1C! In this version, we not only supplemented the figure legends but also provided the expression of *TEK* and *CalS5* in three *pat* alleles as reviewer suggested (Figs. S1 and S5). The results showed that the expression level of *CalS5* is largely reduced in *pat* alleles, which similar with our previous data. Thanks for pointing out our mistakes!

2) We all know that different transgenic alleles could possibly cause developmental defects in different levels, as results of different expression level. Therefore, I would suggest the authors to show the relative expression of TEK in pat-1, pat-2, and pat-3, and clearly state the allele used in each following study but not only address ‘pat mutant’.

Answer: In this work, we first transformed the *pAMS:TEK-GFP* construct into the WT plants, finding that T1 generation lines are all male sterile only they contain the insertion by PCR identification (Fig. S1A). To avoid that the *pat* phenotype comes from the different transgene insertion or expression levels, we then repeated this transformation several times, and found the T1 generation with transgene insertion all showed the male sterile phenotype. We also performed the expression analysis and cytological observations in different *pat* lines among T1 generation, the results are also similar (Figs. S1-3). Additionally, we used WT pollen to cross-fertilize several independent *pat* stigmas for producing their F1 seeds. In these F1 generation, we used PCR to confirm again that plants show male sterility with insertion while others show normal without insertion. Thus, based on these results, we think that the phenotype in *pat* lines did result from the premature expression of TEK in tapetum and we uniformly name them as *pat*.

3) I could not find any indication of which statistical tests were used and which level of confidence are applied in Figure 4 and Figure 6.

Answer: These are our mistakes! We revised them in this manuscript and provided the statistical data in S2 Table, thanks!

3. Given the pat mutant showed reduced CalS5 expression and increased H3K9Me2 in CalS5 promoter, we would expect to see the increased CalS5 expression and decreased H3K9Me2 in tek mutant. I would invite the authors to test these to improve the precision and impact of these analyses.

Answer: According to our data, it’s reasonable to think that the increased *CalS5* expression in *tek*. Thus, we performed the *in situ* hybridization to detect whether the expression of *CalS5* are changed in *tek* (Fig. 5). The results showed that *CalS5* still highly expresses in the tapetum and microspores of *tek* at stage 8 (Fig. 5I) in contrast with none expression in WT (Fig. 5D). Therefore, it’s clearly showed that the expression level of *CalS5* is increased and expression pattern of *CalS5* is prolonged in *tek* at stage 8 compared with that in WT. To detect the decreased H3K9Me2 in *tek* is a very good suggestion. However, it will take a long time for us to analysis this result, because the materials for ChIP assay need a lot and we usually take three different DNA samples and replicate two times for each qChIP-PCR. Additionally, our major claim is that the correct timing regulation of TEK is required for pollen wall exine patterning. It’s well known that pollen wall regulators are crucial for pollen wall formation, here, we further reveal that the expression pattern of regulator also plays a role in pollen wall patterning, which supported by the existing data. So, in this manuscript we still focus on the effects of TEK temporal regulation to the pollen wall development. Certainly, we really thank for your precious suggestion!

Reviewer #2: The current work has its foundation in an earlier work by Lou et al (reference no 38 in the manuscript). The earlier paper had demonstrated the role of TEK gene in pollen development, wherein a knockout of the gene led to male sterility. This paper also showed that the TEK gene was directly regulated by AMS gene. In the current work the authors analyze the effects of expressing the TEK gene at stages of anther development earlier to that of its normal expression. This they achieve by expressing the TEK gene under the AMS promoter in transgenic Arabidopsis lines. Although the observations are interesting, the conclusions drawn from the observations seem improper.

We appreciate for the reviewer’s recognition for this work.

My major concern is:

The authors report that under the AMS promoter the TEK gene is expressed precociously in stage 5 and 6 in the pat transgenic lines as is shown in Fig 1. An important observation that there seems to be reduction in TEK expression at stage 8 in the pat lines (compare Fig 1E to 1J) is not discussed. Is the pattern presented in the Figure observed in independent transgenic lines developed by the authors?

Answer: It’s possible that the partial thins were peeled off so that it seems to be a reduction of TEK expression at stage 8 in *pat* compared with that in WT. In fact, the hybridization signals in tapetum in *pat* are similar with those in WT (we rearranged the arrow position to better show the results in Fig. 1J). Moreover, we provided the expression of *TEK* in three independent *pat* lines (Fig. S2A-L), showing that expression level of *TEK* is not affected in *pat*.

In this work, we first transformed the *pAMS:TEK-GFP* construct into the WT plants, finding that T1 generation lines are all male sterile only they contain the insertion by PCR identification (Fig. S1A). To avoid that the *pat* phenotype comes from the different transgene insertion or expression levels, we then repeated this transformation several times, and found the T1 generation with transgene insertion all showed the male sterile phenotype. We also performed the expression analysis and cytological observations in different *pat* lines among T1 generation, the results are also similar (Figs. S1-3). Additionally, we used WT pollen to cross-fertilize several independent *pat* stigmas for producing their F1 seeds. In these F1 generation, we used PCR to confirm again that plants show male sterility with insertion while others show normal without insertion. Thus, based on these results, we think that the phenotype in *pat* lines did result from the premature expression of TEK in tapetum and we uniformly name them as *pat*. Therefore, we took several inflorescences from different transgenic lines for *in situ* hybridization (Fig. 1B-K), which were confirmed by PCR identification and all showed male sterility.

This quantitative observation is important to reach conclusions on the observed expression pattern of CalS5 gene in the pat transgenics (Fig 6). It is observed that the main difference in expression of Cals5, between WT and the pat lines lies in stage 8 of anther development. The expression of CalS5 is prominent in S8 in pat lines while there seems to be no expression in wild type (compare Fig 6D and 6I). There is no difference in expression between WT and pat lines in the earlier stages. The authors also record this observation (lines 241 to 246). However, they tend to argue that “Thus, considering that TEK-GFP expressed precociously, we supposed that premature TEK decreases CalS5 expression in pat anther”. The observation however, tends to show that the precocious expression of TEK in stage 5 and 6 does not change the expression pattern of CalS5, which they also mention in the text. However, the down regulation of TEK at stage 8 in pat lines leads to expression of CalS5 gene at this stage Thus, while TEK does seem to down regulate CalS5, the observations tend to show that it happens at a later stage (8) rather than at stages 5 and 6.

The results section concludes by saying “All these results suggested that premature appearance of TEK represses CalS5 expression via modulating the H3K9 demethylation in pat” (Line 271)

However, the initial part of discussion (line 281) the authors mention that “In the present study, we revealed that TEK specifically represses the expression of CalS5 in wild type anther after the tetrad stage (Fig 5I)”, which seems to be correct but not in line with the arguments given in the results section. The authors then discuss that the phenotypic outcome is because of the down regulation of Cals5 in the in the earlier stages which is not supported by the presented observations.

It is suggested that the authors reanalyze their observations and discuss it with more clarity.

Answer: In the former version, we only detected the transcript levels of *CalS5* in WT and *tek* not in *pat* anther. In this version, we modified the imagines with labeling to better present our data. Additionally, we also performed the *in situ* hybridization to show the *CalS5* expression in three independent *pat* lines (S5 Fig). In contrast to the WT anther, the hybridization signals in *pat* lines were reduced in tapetum (S5 Fig). We hope these results can better explain our main conclusion.

Other points are:

i. Introduction on pollen development can be shortened. The introduction could concentrate more on the role of TEK in development of pollen, highlighting the initial study by Lou et al. The reason for asking the present question should also be mentioned in the introduction.

Answer: We shortened and modified the introduction in this version, thanks!

ii. As the TEK gene was driven by the promoter of the AMS gene, the spatial and temporal expression pattern of the AMS gene needs to be introduced. It would have been nice if AMS expression patterns had been shown in WT as well as in the pat transgenic lines, as perturbations in AMS expression can also lead to male sterility as has been observed in the earlier work. Thus it needs to be demonstrated that in the transgenic lines developed there is no changes in the AMS expression pattern.

Answer: It’s a very good suggestion that to introduce the expression pattern of the *AMS*. We supplemented this content in our introduction. Moreover, to detect whether *AMS* expression pattern is disturbed or not is also very helpful for our manuscript. Thus, we tested the *AMS* expression levels in three *pat* lines and found *AMS* transcript wasn’t affect in those lines (Fig. S4I). We also performed the *in situ* hybridization to examine the expression pattern of *AMS* in *pat-3*. The results showed that the expression pattern of *AMS* in *pat-3* is similar with that in WT (Fig. S4A-H), excluding the possibility that the changed *AMS* expression leads to the *pat* phenotype. Thanks!

iii. The manuscript mentions that because AMS is upstream to TEK (line 107), the expression of AMS should be at an earlier stage. A gene being upstream to another gene, does not ensure its expression at an earlier stage. It could express at the same stage but as a cascade.

Answer: That *AMS* initially expresses in tapetum at stage 5 has been shown in Zhu *et al*., 2011. Compared with the *TEK* expresses in tapetum at stage 7 in WT, it can be found that *AMS* expresses earlier than *TEK*. Therefore, we used the *AMS* promoter and tried to early initiate TEX expression in tapetum. The reviewer’s opinion is right that our statement is inaccurate. We revised this part for better explaining our data, thank you.

iv. The details of the constructs used for developing transgenic lines should be presented. Line 130: It is not clear what is meant by lines that did not carry insertion: were they escapes during hygromycin selection or they carried the hptII but lacked the AMS:TEK transgene?

Answer: They did escape during hygromycin selection. So, after transformation, we always checked each transformants by PCR identification combing with their phenotype to avoid mixing the false positive lines. We also provided the details of the constructs in methods as reviewer suggested.

v. Line 138: In the wild type anther TEK expression is observed in S7 and S8 (both in situ and GFP in TEK:TEK-GFP lines) and not from S6-8 as mentioned in the text.

Answer: This is our mistake! We revised this in the text, thanks!

vi. How many independent transgenic lines were analyzed for spatial expression of the different genes by in situ hybridizations? Is the presented pattern observed in all the lines tested or only in a subset?

Answer: Three independent T1 transgenic lines were analyzed for cytological observations (Figs. S1-3, 5), which are similar with each other. Moreover, we used WT pollen to cross-fertilize several independent *pat* stigmas for producing their F1 seeds. In these F1 generations, combing with PCR identification, we confirmed again that all plants show male sterility with insertion while others show normal without insertion. Based on these results, we think that the phenotype in *pat* lines did result from the premature expression of TEK in tapetum. Therefore, the materials we took for *in situ* hybridizations (Fig. 1G-K) are the sets which consist of the male sterile plants with transgene insertion tested by PCR identification and phenotype observation. Additionally, the presented pattern shown in Fig. 1 was similar with that in Fig. S2A-L.

vii. The reduction in expression of the TEK gene at stage 8, in pat lines as compared to WT should be critically evaluated. The possible reasons for the same should be discussed.

Answer: Actually, the hybridization signals in *pat* are similar with those in WT (we rearranged the arrow position to better show the results in Fig. 1J). Moreover, we provided the expression pattern of *TEK* in three independent *pat* lines (Fig. S2A-L), the results presented in Fig. S2D, S2H, S2L, Fig. 1J are similar.

viii. In experiments with qRT-PCR, the number of transgenic lines analyzed should be clearly mentioned. Currently it mentions that experiments were repeated three times. Are these replications from different RNA samples or three times from the same RNA sample.

Answer: For qRT-PCR, the materials are the sets which consist of the male sterile plants with transgene insertion tested by PCR identification and phenotype observation. In this version, we also provided the qRT-PCR data from the independent transgenic lines (Fig. S1C). All the experiments with qRT-PCR were performed with three replications from different RNA samples and each sample were repeated with three times by a pair of primers.

ix. The evidence or literature showing that β-tubulin is an appropriate gene for normalizations in the stages studied should be mentioned.

Answer: We added the literature in the text, thanks.

x. The Ct values corresponding to the relative values presented in Fig. 4 could be included as a supplementary table. This helps in evaluating the strength of the presented data.

Answer: We provided these Ct values in S2 Table, thanks.

xi. In experiments of qRT-PCR or ChIP, it seems that an inflorescence has been used a sample. This would mean that buds of different stages would be present and thus conclusions from the observations become weak. It is thus important that results of at least three independent transgenic lines are presented to see the general trends.

Answer: Whatever for qRT-PCR or ChIP experiments, we discarded the buds after stage 12 and tried to decrease the buds which are not involved in the expression period we interested in. In this work, we also provided the cytological observations and expression analysis in the independent transgenic lines, which all showed the consistent results (Figs. S1-3, 5) and this time we also performed the EMSA assay to further confirm our conclusion (Fig. 6C). Therefore, we believe that our data can truly present the general trends.
