# Supplementary material for: The temporal regulation of TEK contributes to pollen wall exine patterning
Source: PLoS Genet. 2020 May 14;16(5):e1008807. doi: 10.1371/journal.pgen.1008807 (PMC7252695; doi:10.1371/journal.pgen.1008807)
Supplement: Attachment — Submitted filename: response to reviewers conmments.docx [file pgen.1008807.s011.docx]

Comments to the Authors:

Please note here if the review is uploaded as an attachment.

Reviewer #1: no more comments

Reviewer #2: Comments on the revised version:

i. The authors have presented results of three independent transgenic lines in the revised manuscript. Although the authors uniformly call the three independent lines as pat lines, I feel that they should be presented as pat-1, pat-2 and pat-3. Further, data from one of the lines say pat-1 is presented in the main text and those of the additional 2 lines in the supplementary.

Answer: We followed the reviewer’s suggestion to present these lines as *pat-1*, *pat-2* and *pat-3* in the text. Based on the phenotypic analyses with independent *pat* lines (shown in the supplementary), we confirmed that the premature expression of TEK in tapetum leads to the phenotype in *pat* lines. Therefore, we used the materials which consist of the male sterile plants with transgene insertion for analysis and we uniformly name them as *pat*. So, we cannot say the data in the main text from one individual line and that’s the reason why we supplemented three independent lines instead of two in the supporting information.

ii. The gene that has been amplified to check the presence of transgene in supplementary figure 1, should be mentioned in the legends. Why does one of the WT plants in this figure have a similar amplification profile as the transgenic lines?

Answer: We modified the legends for supplementary figure 1. It’s true that there is an amplification profile in one WT plant which close to the fragments amplified by primers PAMSJD-F and CTEKJD-R. However, this DNA fragment actually is about 800-bp which is different from the target fragments with 694-bp. Furthermore, it can be found that there are some non-specific DNA bands in those WT plants and this ‘similar amplification’ also appears in the fifth lane which just a little bit weak. So, we supposed this fragment is produced by the non-specific amplification during PCR process.

iii. While the text (line 103) mentions that AMS transcripts are observed in anthers from stage 5, supplementary figure 4 shows results from stage 6 only. If possible the expression at stage 5 should also be included.

Answer: We provided the expression at stage 5 in the supplementary figure 4, thanks!

iv. I feel lines 126 to 128 should be rewritten as: "Of the 26 transgenic lines, 21 were observed to be male sterile and 5 lines were fertile. All male sterile lines carried the transgene as assessed by amplification of (mention the gene that was amplified) fragment while fertile events did not show amplified product." The lines marked as WT in the amplification profile in supplementary figure 1 are actually not untransformed wild type plants but those that probably were male fertile and also lacked the amplification product. This needs to be clearly mentioned.

Answer: We rewrote this sentence in the text and also renamed the lines without the amplification product as FP (Fertile Plants) which distinguish from the WT plants. Thanks for these suggestions!

v. Lines 129 to 134 should be written with clarity, taking into consideration the first point.

Answer: We presented these independent lines as *pat-1*, *pat-2* and *pat-3* in the text, thanks!

vi. The section on CalS-5 expression in wild type, tek mutant lines and pat transgenic lines should be clearly written. Figure 5 should also include the hybridization profile of one of the pat lines. This will help a reader get a comparative view and understand the conclusion easily.

Answer: We modified this paragraph in the text and provided the hybridization profile of *pat* lines, thanks!

vii. In the gel retardation assay, the meaning of unlabeled competitor probe is not clear. Is it unlabeled P11 fragment? If yes, then one cannot make any conclusions on biding specificity. Further, the conclusions are drawn based on the second band (from top), without commenting on the band which represents maximum retardation. With the observed % of fragments that are retardation, one would have expected no retardation in 300 fold excess.

Answer: Yes, we used the unlabeled P11 fragment as the competitor probe. This competitor probe contains the same P11 sequence but unlabeled with 5’ biotin. In the EMSA assay, using the unlabeled competitor probe is a general way to confirm the binding specificity (Xu et al., Plant Cell 2014; Ferguson et al., New Phytologist 2017; Yang et al., Plant Physiology 2013). When adding this kind of competitor probes to the reaction mixture, the corresponding band shift will be reduced in a concentration-dependent manner. In our results, the signal shift of second band is gradually reduced following the increased fold of competitor probes and almost is prevented by competition from 300-fold excess. Therefore, we think this result shows the ability of TEK to bind to the P11 region. We also marked the shifted band in the figure 6 and modified the figure legends, thanks.
