# Supplementary material for: The temporal regulation of TEK contributes to pollen wall exine patterning
Source: PLoS Genet. 2020 May 14;16(5):e1008807. doi: 10.1371/journal.pgen.1008807 (PMC7252695; doi:10.1371/journal.pgen.1008807)
Supplement: Attachment — Submitted filename: Response to Editor and Reviewers.docx [file pgen.1008807.s013.docx]

**Discussion**

Both primexine formation and membrane undulation are involved in pollen wall ornamentation [56]. Thin or absent primexine is usually found in callose deficient mutants, such as *cals5*, *cdkg1* and *arf17* mutants [43, 48, 50], suggesting that callose may provide a surface against which primexine is deposited or act as a source of glucose for primexine formation. In *pat* anthers, the primexine matrix still thickens normally at stage 7 (Fig 3Q-3S), and the expression of genes required for primexine formation is not affected (Fig 4B), suggesting that the reduced callose wall of *pat* may affect primexine for exine patterning through other routes. The primexine is described to be a polysaccharide material [15, 26], and the mixture of these polysaccharides is not stable [57]. Recently, it has been reported that polysaccharide materials tend toward demixing in the primexine, leading to spatially modulated phase separation. When primexine separation is in different states, it will form different templates for pollen wall deposition. It is speculated that membrane undulation in the vicinity of the callose wall induces this phase separation, and that components, including sporopollenin polymers and cellulose fibrils, arrest the phase separation [58]. We speculate that the reduced callose wall, straight membrane, and early appearance of sporopollenin precursors in *pat* may alter the phase-separation process in the primexine, leading to a change in exine patterning.

15.	Heslop-Harrison J. Pollen wall development. The succession of events in the growth of intricately patterned pollen walls is described and discussed. Science. 1968;161(3838):230-7. PubMed PMID: 5657325.

26.	Li WL, Liu Y, Douglas CJ. Role of Glycosyltransferases in Pollen Wall Primexine Formation and Exine Patterning. Plant Physiol. 2017;173(1):167-82. doi: 10.1104/pp.16.00471. PubMed PMID: 27495941; PubMed Central PMCID: PMCPMC5210704.

57.	Agoda-Tandjawa G, Durand S, Gaillard C, Garnier C, Doublier JL. Properties of cellulose/pectins composites: implication for structural and mechanical properties of cell wall. Carbohydrate Polymers. 2012;90(2):1081-91. doi: 10.1016/j.carbpol.2012.06.047. PubMed PMID: 22840043.

**Fig 5. *CalS5* expression patterns in the wild-type, *pat* and *tek* anthers.** Expression of *CalS5* in microspore mother cells, tetrads and tapetum was tested by RNA in situ hybridization in WT (A–D), *pat* (F-I) and *tek* anthers (K-N) at stages 5-8 using an antisense probe. According to the stages at which *CalS5* reaches its peak with an antisense probe, its transcript was observed with a sense probe in WT anthers at stage 6 (E), *pat* anthers at stage 5 (J), and *tek* anthers at stage 8 (O). MMC, microspore mother cell; MC, meiocytes; T, tapetum; Tds, tetrads; Msp, microspore. Scale bars, 20 μm.
